# Design and Investigation of a Passive-Type Microfluidics Micromixer Integrated with an Archimedes Screw for Enhanced Mixing Performance

**DOI:** 10.3390/mi16010082

**Published:** 2025-01-12

**Authors:** Muhammad Waqas, Arvydas Palevicius, Vytautas Jurenas, Kestutis Pilkauskas, Giedrius Janusas

**Affiliations:** Faculty of Mechanical Engineering and Design, Kaunas University of Technology, 51424 Kaunas, Lithuania; vytautas.jurenas@ktu.lt (V.J.); kestutis.pilkauskas@ktu.lt (K.P.); giedrius.janusas@ktu.lt (G.J.)

**Keywords:** micromixing, Archimedes screw, microfluidics, mixing index, performance index

## Abstract

In recent years, microfluidics has emerged as an interdisciplinary field, receiving significant attention across various biomedical applications. Achieving a noticeable mixing of biofluids and biochemicals at laminar flow conditions is essential in numerous microfluidics systems. In this research work, a new kind of micromixer design integrated with an Archimedes screw is designed and investigated using numerical simulation and experimental approaches. First, the geometrical parameters such as screw length (*l*), screw pitch (*p*) and gap (*s*) are optimized using the Design of Expert (DoE) approach and the Central Composite Design (CCD) method. The experimental designs generated by DoE are then numerically simulated aiming to determine Mixing Index (*MI*) and Performance Index (*PI*). For this purpose, COMSOL Multiphysics with two physics modules—laminar and transport diluted species—is used. The results revealed a significant influence of screw length, screw pitch and gap on mixing performance. The optimal design achieved is then scaled up and fabricated using a 3D additive manufacturing technique. In addition, the optimal micromixer design is numerically and experimentally investigated at diverse Reynolds numbers, ranging from 2 to 16. The findings revealed the optimal geometrical parameters that produce the best result compared to other designs are a screw length of 0.5 mm, screw pitch of 0.23409 mm and a 0.004 mm gap. The obtained values of the mixing index and the performance index are 98.47% and 20.15 Pa^−1^, respectively. In addition, a higher mixing performance is achieved at the lower Reynolds number of 2, while a lower mixing performance is observed at the higher Reynolds number of 16. This study can be very beneficial for understanding the impact of geometrical parameters and their interaction with mixing performance.

## 1. Introduction

In recent years, microfluidics technology has emerged as a pivotal interdisciplinary field, receiving substantial attention across numerous biomedical applications, including mixing, sorting, separation, detection and reaction [1,2,3]. Microfluidics technology employs microchannels to handle and control tiny volumes of samples precisely and efficiently, revolutionizing biomedical research and clinical diagnostics. In addition, microfluidics is a rapidly growing technology due to its inimitable properties, such as elevated surface-area-to-volume ratios and enhanced heat and mass transfer, driving innovations across numerous scientific and commercial domains, offering high efficiency and cost-effectiveness in various biomedical applications [4,5]. Among various microfluidics systems, mixing is one of the most intriguing processes, where microchannels within a tiny dimension, ranging from micrometers to millimeters, aim to achieve a homogeneous mixture [6]. Mixing is normally governed by diffusion instead of turbulence due to its low Reynolds number and laminar flow conditions. Additionally, extensive research has been conducted on various kinds of micromixers and noted two main types, active and passive, depending on their actuation field [7,8,9].

In active-type micromixers, some external energy sources such as the electric field [10], magnetic field [11], thermal field [12], pressure field [13] and acoustic field [14,15] are used to enhance the mixing of two heterogeneous fluids, facilitating fluid perturbation and strengthening contact areas, leading to higher efficiency. These kinds of active sources introduce disturbance in the fluid flow streamlines, which increases species transport during the mixing process [16]. Although active micromixers provide certain advantages in the rapid and controllable mixing of fluids, they also have numerous drawbacks. In other words, an active micromixer requires higher energy usage, particularly during prolonged operation, leading to complicate and increased experimental costs [17]. Additionally, active micromixers often need complicated laboratory setups and control systems such as acoustic actuation systems, electrode arrays and magnetic controllers etc. [18]. Sintayehu Assefa Endaylalu et al. [19] investigated Y-shaped microchannels with acoustic streaming by adding a triangular structure via a numerical simulation approach. The results showed that the mixing performance increased with the addition of the triangular structure at the junction point of the Y-shaped microchannel and sidewall of the channel because of the production of acoustic streaming at a lower inlet flow velocity. Habib Jalili et al. [20] investigated the optimization of impactful parameters and their effects on the mixing performance of an active kind micromixer. The outcomes revealed the electroosmotic force which perturbed the parallel streamlines in the laminar flow, leading to higher-order distortions. Yanwen Gong et al. [21] investigated the contraction—expansion microchannel caused by AC electroosmotic active actuation using a numerical simulation approach. The authors investigated the concentration distribution and flow field in the microfluidics microchannel and observed the two circulating flow field regimes and four rotating cortices generated in the expansion chamber by the applied AC electric field [22].

Conversely, the passive-type micromixers do not require any external energy source but function primarily on flow manipulation by specific channel design configurations and features [23]. These features are particularly aimed at attaining the repeated splitting and recombination [24,25] of the flow which induce the fluid rotation and generate micro-vortices as well as induce chaotic mixing [26]. Furthermore, mass transfer is also governed by passive-type micromixers using molecular diffusion and convection, leading to a reduction in diffusion paths. Remarkable theoretical, experimental and numerical experiments using various passive-type micromixers can be found in [27,28,29]. ZhenghaoWang et al. [30] investigated a 3D-printed micromixer which excelled in the diffusion process and improved mixing performance at lower Reynolds number by employing a design that splits, stretches and then recombines fluid streamlines. Ekta Tripathi et al. [31] proposed the following three novel spiral-type micromixers: a meek spiral micromixer; an SE type micromixer, premeditated by changing the center part of the spiral design with straight walls; and an RS micromixer, produced by designing the whole spiral part with straight walls. Kevin Ward et al. [32] examined a novel approach used to increase the mixing performance within the micromixer domain. The authors used ridges and slanted walls and changed the different geometrical parameters in order to evaluate the mixing performance by using a numerical simulation approach. Jae Bem You et al. [33] projected and evaluated a Y-shaped turbulent kind micromixer that is produced using PDMS and glass substrate to evaluate the mixing performance through numerical simulations and experimental techniques. Min Xiong et al. [34] carried out a topology optimization to enhance the mixing performance based on the principle of the Tesla valve. Lili Zou et al. [35] introduced an innovative micromixer design and investigated its mixing efficiency in fluid dynamics.

Experimental optimization is an effective approach which has gained attention among researchers in the cases where the interactions of all variables are considered simultaneously. Design of Experiment (DOE) is one of the most powerful tools for optimizing purposes, providing valuable insights into how design and operation variables interact and impact the entire system [36,37]. DoE has been widely used in numerous domains such as biomedicine, materials science and chemistry [38]. In addition, the main advantages of integrating DoE into a micromixer design is the generation of response surfaces which can be useful for exploring the relationship between input variables and determine the significant changes in the output response. These output responses can also serve as an optimization approach in order to achieve optimal design. This approach has been successfully applied to numerous engineering domains [39]. The mixing with Response Surface Methodology (RSM) has also been employed to achieve optimal micromixer design [40]. Fabricating microfluidics microchannels is a process that involves various steps such as substrate preparation, mold creating, mold preparation, channel creating and bonding between substrate and device to fabricate microchannels completely. Thanh Qua Nguyen et al. [41] used a multi-depth approach to create circular-shaped PDMS microchannels. They used inflated air pressure to deform a particularly cured PDMS with a simple bench-top apparatus. P. Gaso, D. Jandura et al. [42] proposed an unconventional approach where a photoresist of an appropriate viscosity can be drawn into thin fibers of cylindrical shape. They also presented a distinctive technique of pattering the prepared photoresist fiber by using optical interference lithography. Kaori Uehara et al. [43] used the Mosquito method to create circular-shaped microchannel with 3D lattice arrangements. Thanh-Qua Nguyen et al. [44] used a novel fabrication process of creating circular-shaped PDMS microchannels using air pressure to define the deformation of partially cured PDMS surfaces.

The mixing index and pressure drop are the most significant parameters for measuring the mixing performance when two different heterogeneous fluids pass through the microchannel [45]. There are numerous parameters, including as flow rate, viscosity, shear rate and other geometrical parameters, that impact overall mixing efficiency in microfluidics systems [46]. Researchers have explored several micromixer geometries but the most common are Y-shape and T-shape geometries, aiming for enhancements in the interfacial area to increase mixing performance [47]. Xin Dong et al. [48] proposed an innovative T-shape micromixer, including the impact of Newtonian and non-Newtonian fluids, to examine the use of topology optimization in order to enhance mixing quality with a lower pressure drop. The results revealed mixing efficiencies of 93.90% for a Newtonian fluid with Reynolds number 8 and 93.84% for a non-Newtonian fluid with Reynolds number 0.24, respectively. Muhammad Waqas et al. [49] proposed an innovative design of a microfluidics micromixer, which included different micropillar shapes such as a circular shape, hexagonal shape, and blade shape inside the microchamber. They revealed the maximum mixing performance occurs with blade-shape micropillars. An-Shik Yang et al. [50] investigated a new higher performance Tesla-shape micromixer to comprehend the effective mixing phenomena in microfluidics systems for biomedical applications. For this purpose, they used both computational and experimental approaches to examine the mixing characteristics in the design and development of a 3D Tesla-type micromixer. The results showed an excellent mixing performance for the proposed micromixer with a lower pressure drop. Additionally, more details about passive-type micromixers with different geometrical features are listed in Table 1.

According to the currently available literature. It is observed that mixing is the most important phenomenon for various applications, especially in the biomedical domain. Passive strategies such as changing geometrical configurations and design parameters are used by many researchers to reduce energy consumption and improve reliability. Commonly micromixer designs, such as Y-shape; T-shape; Tesla-shape; microchamber with circular, hexagonal and blade shape micropillars; serpentine-shape; spiral-shape; flower-shape and plate column tray-inspired microchannel micromixers, is used by most researchers. Moreover, it has also been observed that much less work is carried out on microfluidics micromixers integrated with an Archimedes screw for enhanced mixing performance. The mixing efficiency ranging from 80% to 95% is normally observed by many researchers and very few micromixer designs have achieved a mixing efficiency of up to 97%. The proposed micromixer design integrated with an Archimedes screw provides the significant advantages of higher mixing efficiency, lower pressure drops, mitigation of the risk of clogging, low energy consumption and rapid mixing compared to previous studies.

This work proposes a novel microfluidics micromixer integrated with an Archimedes screw with different geometrical and operational parameters for enhanced mixing performance while sustaining a low pressure drop. The geometrical parameters, such as the length of the screw (*l*), pitch of the screw (*p*) and the gap (*s*) between the screw and the inner surface of the channel, were optimized using DoE. The mixing index (*MI*), pressure drop (Δ*P*) and performance index (*PI*) were used to measure the mixing performance of the micromixer. The numerical simulations were performed on 17 sets of micromixer designs, produced by DoE to compute the mixing performance, pressure drop and performance index. After that, numerical simulations with different Reynolds numbers were conducted on the optimized micromixer design with the highest mixing performance. Moreover, an experimental study was also conducted to validate the numerical simulation results. For this purpose, the optimized micromixer design was scaled up and fabricated using a 3D printing approach. Finally, a comparison study was also conducted between the optimized model and the scaled-up model, both numerically and experimentally.

## 2. Materials and Methods

### 2.1. Micromixer Design and Performance Parameters

**Figure 1:**
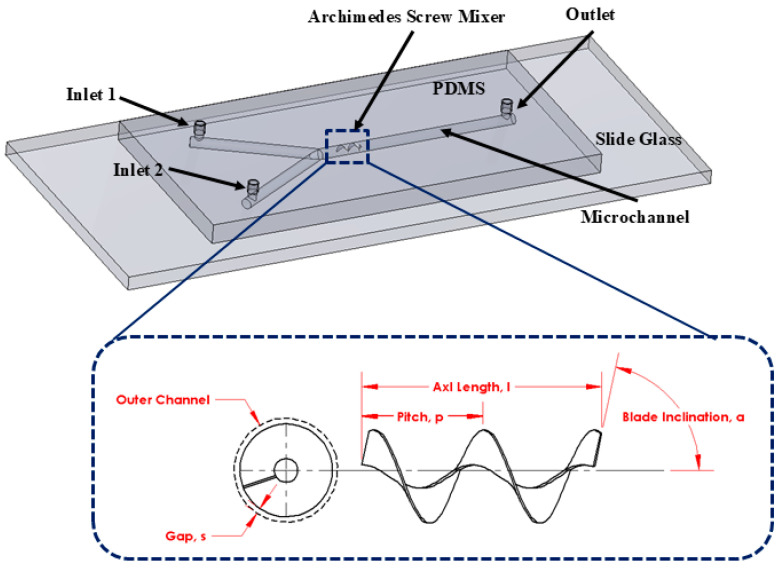
Schematic diagram of microfluidics micromixer integrated with an Archimedes screw.

Figure 1 illustrates the schematic diagram of the Y-shape microfluidics micromixer integrated with an Archimedes screw with detailed geometrical dimensions. The proposed micromixer comprises two inlets where two different heterogeneous fluids are introduced using a syringe pump and one outlet where mixed fluid is collected. An Archimedes screw is placed inside the circular-shape microchannel, considered as a mixing unit, which converts the input laminar flow into spirally moving rotational flow. This means that the screw shape turns the input laminar fluid flow to a continuously rotating flow in a 3D flow trend, causing involute flow velocity variations. This allows the flowing fluid to experience the advantages of chaotic mixing, including varied flow directions as well as diffusive mixing produced by the enhanced contact area. Consequently, the mixing performance can be significantly increased by integrating an Archimedes screw component inside the microchannel.

Moreover, the performance of the micromixers is assessed in terms of the mixing index (*MI*), pressure drop (Δ*p*), performance index (*PI*) and the concentration of two different heterogeneous fluids at the outlet of the micromixer. The mixing index is calculated through a statistical approach, computing the concentration at five different cut planes along the microchannel length based on the standard deviation of heterogenous fluid species using the following mathematical relations [30].

(1) $$MI=1-\sqrt{\frac{\sigma^{2}}{{\sigma^{2}}_{\max}}}$$

(2) $$\sigma =\sqrt{\frac{1}{n}\sum_{i=1}^{n}(c_{i}-\overset{-}{c})}$$

(3) $$\sigma_{\max}=\sqrt{\overset{-}{c}(1-\overset{-}{c}})$$

where σ represents the standard deviation while ci describes the concentration species of the two fluids; $\overset{-}{c}$ shows the mean value of concentration of the two different fluids and $\sigma_{\max}$ describes the maximum standard deviation at a certain section plane across the microchannel. In addition, the range of the mixing index is from 0 to 1, where a higher mixing index value shows a greater mixing performance. A mixing index value of 1 indicates that the fluid is completely mixed, while 0 represents the lack of mixing or the complete separation of the two different fluids, likely due to their heterogenous fluid properties. Moreover, a high-quality micromixer comprised both higher mixing efficiency and lower pressure from the inlet and outlet sections of the microchannel. Thus, the performance index (*PI*) is used to measure the ability to obtain efficient fluid mixing at a lower pressure drop [55]. *PI* is generally defined as the ratio of the mixing index and pressure drop as described by following mathematical equation.

(4) $$PI=\frac{MI}{\Delta p}$$

### 2.2. Experimental Design and Statistical Analysis

Response Surface Methodology (RSM) is employed to investigate the impact of independent variables such as the screw length (*l*), screw pitch (*p*) and gap (*s*) between the screw and the inner microchannel surface on response variables such mixing index and performance index. Table 1 represented the RSM design with coded and uncoded levels. For this purpose, a Central Composite Design (CCD) with three levels is utilized as the optimization tool in the RSM approach to explore the influence of multiple independent variables on the response [59]. The investigation contained twenty-one micromixer designs comprising six axial points, eight factorial points and six central points, which were performed randomly according to CCD, as summarized in Table 2. Because five common design parameters are observed in CCD experiments, a total of 17 different designs were simulated. The total numbers of experiment runs are determined by the following equation.

N = *k*^2^ + 2*k* + *c_p_* (5)

N = 9 + 6 + 6 = 21

where *k* describes the number of independent variables, *c_p_* is the total numbers of central points in CCD and N is the overall number of experiments runs to be performed for optimization purposes.

Furthermore, the quadratic model is used to examine the relationship between independent and dependent variables [60]. The quadratic model is suitable and efficient for acquiring the linear as well as non-linear behavior of the independent and response variables. Additionally, quadratic models play an important role in the investigation of the interaction and curvature influences which frequently exist in micromixer designs, where geometrical parameters significantly affect the overall mixing performance. A second order quadratic equation is used to reveal the predicted responses (mixing index and performance index) as functions of an independent variable, given by following mathematical relationship.

(6) $$\begin{array}{l} Y=b_{o}+b_{1}X_{1}+b_{2}X_{2}+b_{3}X_{3}+b_{11}X_{1}^{2}+b_{22}X_{2}^{2}+b_{33}X_{3}^{2}+b_{12}X_{1}X_{2}+b_{13}X_{1}X_{3}\\ +b_{23}X_{2}X_{3} \end{array}$$

where *Y* illustrates the response values; *bi*, *bii* and *bij* describe the linear, quadratic and interactive coefficient values, respectively; while *bo* is the coefficient constant. For this purpose, the Design Expert V13 software is used to determine the coefficient values.

### 2.3. Computational Domain and Meshing

**Figure 2:**
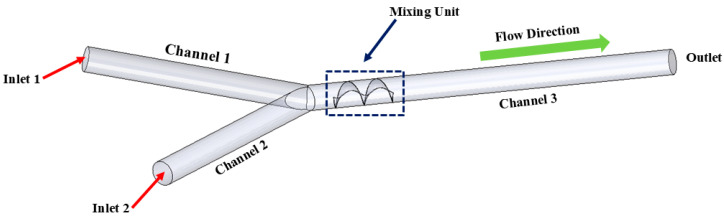
Computational domain for numerical simulations.

**Figure 3:**
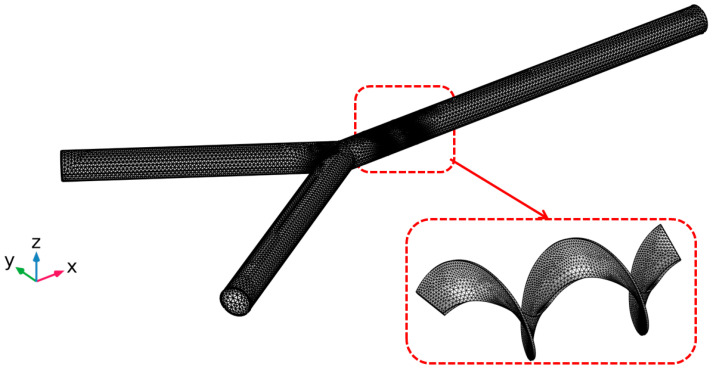
Meshing of micromixer for numerical simulations.

Numerical simulations are conducted on 17 different micromixer designs, obtained by the DoE approach to assess the mixing performance. Figure 2 illustrates the three-dimensional computational domain consisting of two inlets where the two different heterogeneous fluids were injected through a syringe pump at different Reynolds numbers (Re); the mixing unit that generates spirally rotating motion around the screw leading to enhancements in stretching, splitting and mixing the fluid streamlines inside the microchannel; and one outlet to collect mixed sample. The COMSOL Multiphysics tool is used for the numerical simulations of the 17 different micromixer designs. In addition, the meshing of the micromixer design with a 3D tetrahedral element is generated, with total number of elements and nodes being 148,480 and 39,488, respectively. The generated mesh for the micromixer is shown in Figure 3.

### 2.4. Governing Equations

Generally, governing equations typically illustrate the characteristics of fluid flow inside the microchannels. The flowing fluid moves inside microchannel and is considered a laminar that leads to the parallel movement of fluid layers in mixing complexity. This section described a comprehensive explanation and the mathematical relations related to the governing equations used to solve laminar and transport diluted species physics. The incompressible Navier-SATOKES equation and continuity equation are mainly used to describe the fluid flow behavior and hydrodynamics analysis. The incompressible continuity and Navier–Stokes (N-S) equation are used to examine the hydrodynamic and fluid flow analysis. The mathematical relation used for an incompressible continuity equation and the momentum equation are described below [61].

(7) $$\frac{\partial \rho }{\partial t}+\nabla \cdot (\rho v)=0$$

(8) $$\rho \frac{\partial v}{\partial t}+\rho (v\cdot \nabla )v=-\nabla p+\mu \nabla^{2}v+\beta \mu \nabla (\nabla \cdot v)$$

where ρ described the density of fluid, μ represents the fluid dynamic viscosity, β is used for the viscosity ratio and p and *v* define the pressure field and the velocity field, respectively. In addition, the laminar physics-related governing equations are used to describe the flowing fluid behavior in laminar condition. The used governing equations which are related to laminar physics are given by [62].

(9) $$\rho (u2\cdot \nabla )u2=\nabla \cdot \left[-p21+K\right]+F$$

(10) ρ∇·u2=0

where *K* expresses the thermal effect in the flowing fluid and F represents the force exerted by the external volume in the flowing fluid. Moreover, the mixing of two diverse heterogeneous fluids is defined by transport diluted species physics in COMSOL Multiphysics. This model is typically used to define the mixing of two different chemical reactive species. In the current study, the transport diluted-related governing equations are applied to resolve the advection-diffusion phenomena after considering the rate of consumption and production reactants, respectively. The governing equations which are used for the transport diluted species are described below [63].

(11) $$\nabla \cdot j_{i}+u\cdot \nabla c_{i}=R_{i}$$

(12) $$j_{i}=-D_{i}\nabla c_{i}$$

where *c* shows the concentration of different species, *D* describes the diffusion coefficient of fluids, *R* is the rate of reaction for two different species and *j* represents the factor of mass flux which defines the diffusive flux vector.

### 2.5. Numerical Scheme and Boundary Conditions

**Figure 4:**
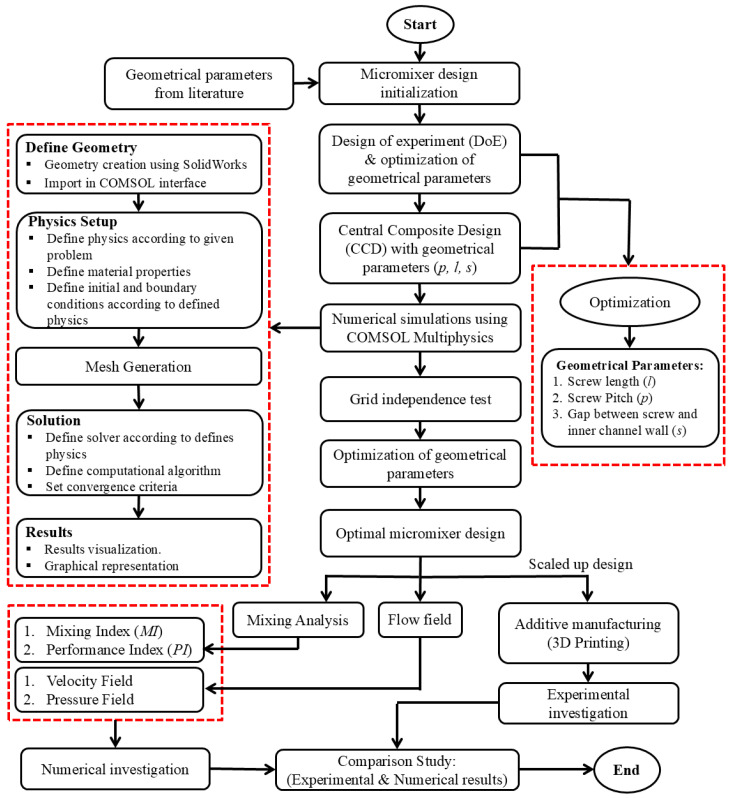
Research and numerical methodology flow chart.

Numerical simulations of the micromixer integrated with an Archimedes screw are performed. For this purpose, COMSOL Multiphysics is used to solve the governing equation using the Finite Element Method (FEM). To perform the mixing simulations, two different physics modules are used as follows: the laminar to address the fluid flow-related governing equations with laminar behavior, and transport diluted species to describe the mixing phenomena and assess the mixing behavior of the two different heterogeneous fluids inside the microchannel. In addition, the microchannel walls are considered solid and stationary with no-slip conditions. Water and ethanol are injected at both inlets as a working fluid which are fully developed and uniform with different Reynolds numbers. The density, dynamic viscosity and diffusion coefficient for the water and ethanol used in the current simulation are 997 Kg/m^3^ and 789 Kg/m^3^, 890 µPas and 1200 µPas, 2.3 × 10^−9^ m^2^/s and 1.2 × 10^−9^ m^2^/s, respectively. The pressure is set to zero at the outlet of the micromixer. Moreover, the concentration of two distinct species is set to be 1 mol/m^3^ at one inlet and zero at the other outlet. Figure 4 illustrates a comprehensive description of research and numerical methodology adopted for the optimization and performance evaluation of the micromixer integrated with an Archimedes screw. The process starts with finding the geometrical parameters such as screw length (*l*), screw pitch (*p*) and the gap (*s*) between the screw and the inner channel wall from the literature, offer a baseline for design initialization. For this purpose, the Design of Experiment (DoE) technique was used, specifically using a Central Composite Design (CCD) for the optimization of the geometrical parameters. The numerical simulations are then performed using COMSOL Multiphysics, starting with defining the geometry using SolidWorks, setting up physics by defining the material properties, boundary conditions and solution scheme etc. A grid independence test is carried out to ensure the reliability and accuracy of the numerical simulations. The findings of numerical simulations including pressure fields, velocity fields as well as performance parameters such as mixing index, pressure drop and performance index are examined and visualized to obtain the optimal design. Moreover, once the optimal micromixer design is obtained, it is scaled up and fabricated using additive manufacturing approaches such as 3D printing and micromachining, etc. The experimental investigations are then performed and validated with the numerical simulation results to examine the micromixer’s performance experimentally.

### 2.6. Grid Independence Test

**Figure 5:**
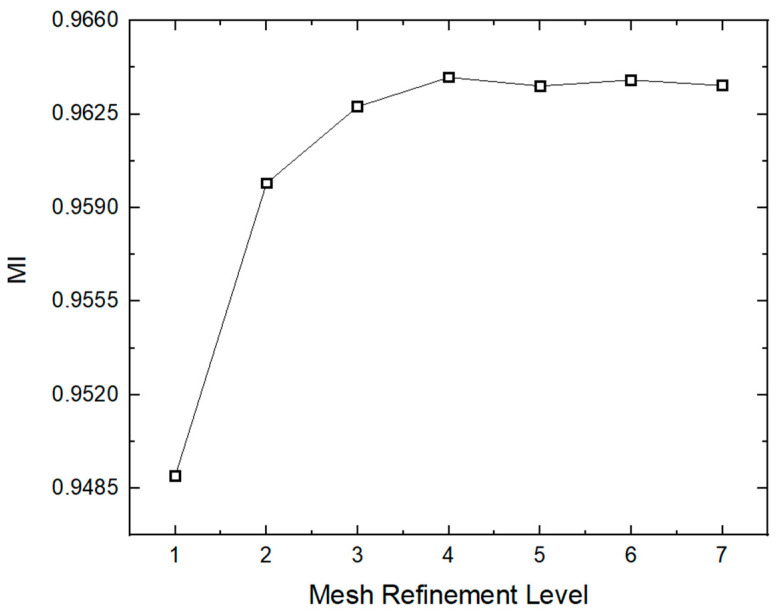
Mesh convergence graph with different mesh refinement levels.

The grid independence test plays an important role in obtaining an accurate solution. The accuracy of the computed results and the computational cost are significantly dependent on the numbers of elements and nodes within the mesh. In this work, the grid independence test is carried out at various mesh refinement levels until a solution stability is reached. Table 3 lists the mesh statistics in detail. To check the grid independence, the mixing index is computed across different mesh refinement levels, as described in Figure 5. The primary aim of this grid independence test is to ensure the accuracy of the simulation solutions. In the current work, a mesh refinement level of 5 is used, comprising 148,480 elements and 39,488 nodes with an average value of skewness of 0.81. It is evident from Figure 5 that there is a minimal difference in the computed results after a mesh refinement level of 5. Thus, a mesh refinement level of 5 is used as the best mesh, which lies within the acceptance range for the current study, offering a balance between solution accuracy and computational efficiency.

### 2.7. Experimental Procedure

**Figure 6:**
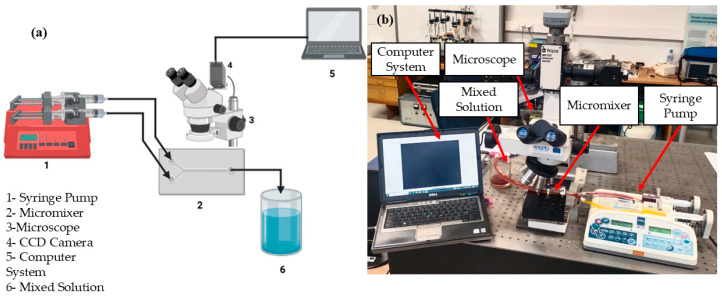
Experimental setup. (**a**) Schematic diagram, (**b**) laboratory setup.

After achieving an optimal micromixer design, the mixing unit was fabricated using an SLA (Stereolithography) 3D printer due to its higher resolution and smooth surface behavior. The mixing unit design was created using SolidWorks to ensure the precise geometrical alignment and dimensional correctness. Photopolymer resin was utilized as a printing material to ensure its biocompatibility and mechanical strength. The printing process involved the layer-by-layer polymerization of the resin using a UV laser, facilitating the fabrication of a complex-feature helical-shape screw and channel wall. The micromixer was carefully detached from the printer platform and resin cleaning was performed using isopropanol and UV curing. Figure 6a,b represents the schematic diagram and experimental laboratory setup used to visualize the mixing mechanism experimentally. For this purpose, two different fluids with different dye colors are used with two different concentrations. The syringe pump Aitecs SEP–21S Plus (Viltechmeda, Vilnius, Lithuania) is used to introduce two different fluids into the microfluidics device to deliver a controlled and continuous flow. The mixing phenomena can be observed at five different section planes through a microscope integrated with a CCD camera, which is mounted on the microscope to visualize the live images of mixing behavior which are directly sent to the computer system for further analysis. The captured images were then transformed to grayscale images with the gray level of fluids to observe the concentration of two different fluids. The empirical relationship was used to convert the captured image intensities to concentration values at five different section planes. Finally, a mixed solution was collected in a container that could be used for further use or testing.

## 3. Results and Discussions

### 3.1. Analysis of Design Optimization

Design optimization is one of the important processes which enable the creation of compact and scalable designs, especially in the microfluidics domain. In the current study, the design optimization of a micromixer integrated with an Archimedes screw is conducted to enhance mixing performance by improving the mixing efficiency and lower the pressure drops. The geometrical parameters such as screw length (*l*), screw pitch (*p*) and the gap between the screw and the inner wall of the channel (*s*) are used for design optimization. The description of the geometrical parameters is shown in Figure 1. The main purpose of this optimization study was to build a scaled-up design that sustained high mixing efficiency while maintaining a low pressure drop which enables the efficient operation at larger outcomes. For this purpose, a Design of Experiment (DoE) with Central Composite Design (CCD) is selected to optimize geometrical parameters. This approach allows for the investigation of interaction between these parameters to attain the optimal design within a defined range. In addition, response surface analysis experiments are also conducted using the mixing index (*MI*) and performance index (*PI*) as resulting responses. First, three geometrical parameters such as screw length of 0.3 mm, 0.5 mm and 0.7 mm, screw pitch of 0.1 mm, 0.15 mm and 0.2 mm and spacings of 0.002 mm, 0.004 mm and 0.006 mm are defined. In addition, a total of 21 design points is generated using DoE, but due to five similar design points, 17 design points are simulated as listed in Table 4. The performance results obtained are taken from the 17 design points for a micromixer integrated with an Archimedes screw at Re = 10. According to the results obtained, the mixing index ranged from 85.26% to 98.47%, while the *PI* ranged from 2.19 to 38.7 Pa^−1^. It is also observed that design point 11 achieved the maximum mixing index and the lowest pressure drop of 2.46 Pa, and the maximum performance index of 38.7 Pa^−1^ was achieved at design point 17. According to the simulated results, the design point 11 is selected as the best design with a 98.47% mixing efficiency and a 20.15 Pa^−1^ performance index, which lies within the acceptable range. Moreover, it was also investigated, using Design-Expert software, whether all three parameters are significantly influenced by the mixing index and performance index. The polynomial statistical models for determining the predicted values of the mixing index and performance index are established using the following relations.

Mixing index (*MI*) = 95.4408 + 0.743659*l* + 2.07305*p* + 0.158027*s* − 2.1125*lp* − 0.02*ls* + 0.01*ps* − 1.49536*l*^2^ + 0.109769*p*^2^ − 0.330404*s*^2^ (13)

Performance index (*PI*) = 8.39338 − 7.31271*l* + 6.22855*p* + 0.0246957*s* − 3.5475*lp* + 0.0525*ls* + 0.095*ps* + 4.03488*l*^2^ + 0.939521*p*^2^ − 0.640863*s*^2^ (14)

**Figure 7:**
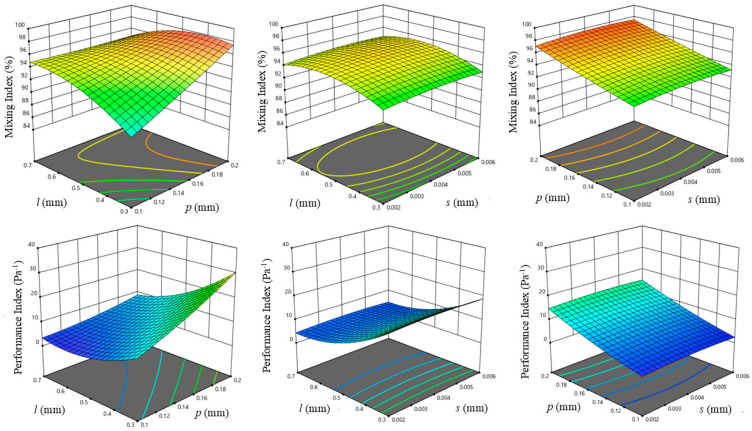
Graphical representation of 3D response surface plot of mixing index and performance index under the interaction of two variables.

Furthermore, the R2 values obtained from the statistical polynomial models for the mixing index and performance index from the analyses are 0.9812 and 0.9845, respectively. According to the statical analysis, the models accurately represent the experimental findings. In addition, these regression models provide the precise prediction of the optimal mixing efficiency of the micromixer. The parameters influencing the mixing index and performance index are screw length, screw pitch and spacing depending on F-values. The impact of interaction between the other two parameters on the mixing index and performance index was investigated by fixing one of the parameters at the central value through the control variable technique. The detailed 3D response surface plots for the mixing index and performance index can be observed in Figure 7.

The multi-objective optimization of the micromixer design was performed using Design Expert V13 software based on single-objective results. The predicted analytical results showed that the optimized geometrical parameters are a screw length of 0.5 mm, screw pitch of 0.15 mm and spacing of 0.004 mm between outer screw surface and inner channel wall, obtaining a mixing index of 97.94% and a performance index of 0.013 Pa^−1^ at Reynolds number 10. In addition, the numerical simulation results of the optimized micromixer achieved using COMSOL Multiphysics 6.1 software produced a mixing index of 98.47% and a performance index of 0.014 Pa^−1^ at Reynolds number 10, which are in good agreement with the theoretically predicted results. After obtaining the optimized geometrical parameters, the device is scaled up for fabrication purposes. The optimized design is used for further numerical simulations at different Reynolds numbers.

### 3.2. Analysis of Mixing Performance

**Figure 8:**
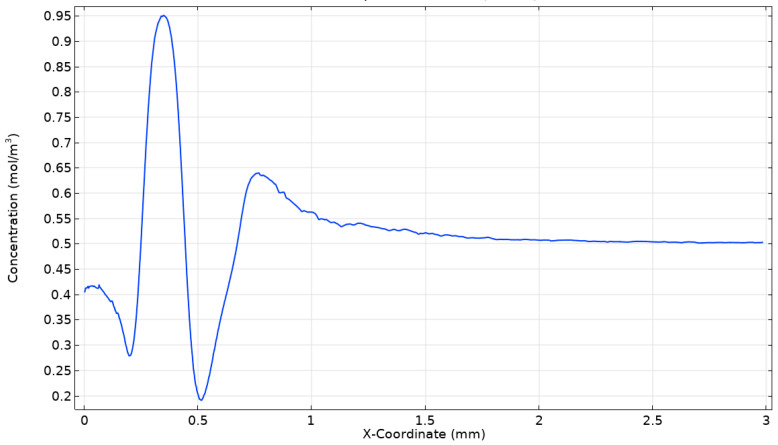
Variation in mixing concentration along the length of the channel at Re = 10.

The mixing evaluation of the optimal micromixer integrated with an Archimedes screw is carried out to examine the mixing performance. The two indicators considered are mixing index and performance index to evaluate the mixing quality. The higher the mixing index and performance index, the greater the mixing performance. First, the concentration distribution along the length of the channel is presented in Figure 8. Two different fluids, water and ethanol, are inserted through two inlets with varying concentrations. The mixing performance enhances as the concentration reaches 0.5 mol/m^3^ at an outlet section of the micromixer. It can be observed from Figure 8 that the concentration changes across the length of the micromixer. At first, a substantial variation in the concentration is observed, revealing the area of mixing activity. As the flow proceeds, the concentration becomes stable and unform, near 0.5 mol/m^3^ towards the outlet of the micromixer.

**Figure 9:**
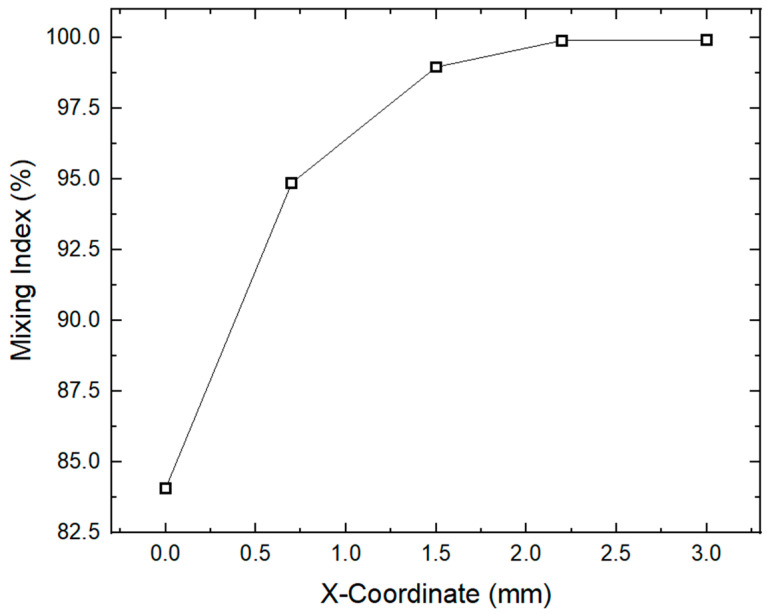
Variation in mixing index along the length of the channel.

**Figure 10:**
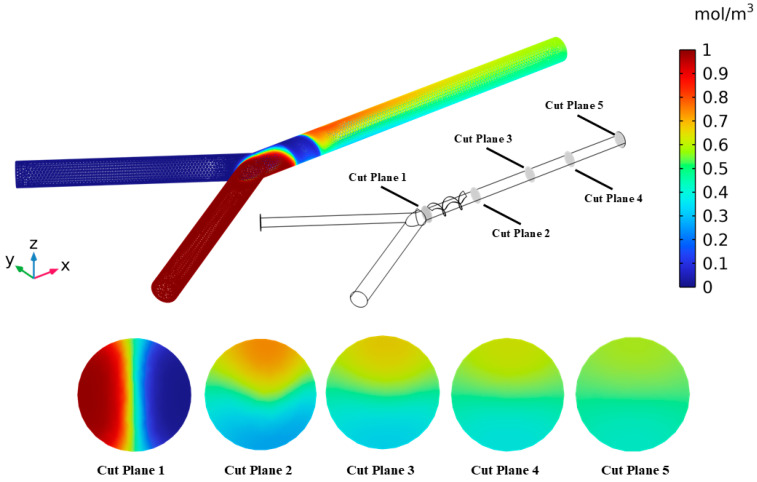
Variation in mixing concentration with five different cut planes.

Similarly, Figure 9 represents the variation in the mixing index along the length of the channel. The mixing index generally measures the degree of homogeneity of the mixture, revealing the complete mixing at a 100% mixing index. It is evident that the mixing index is increased along the length of the micromixer channel. The minimum and maximum values of mixing indexes equivalent to 85.26% and 98.47% are observed, respectively. The initial level of mixing appears at the inlet section, and then, the mixing index increases steadily with the flow progress, showing an improved mixing performance. Moreover, the contour representation of the concentration of the micromixer design at five different cut planes is also shown in Figure 10. The mixing visualization is described by color scale, ranging from red showing—high concentration—to blue—lower concentration. Five different cross-sectional planes are developed along the length of the channel to examine the mixing performance. The cut plane 1 represents the unmixed region before the mixing unit near the inlet section. As the flow proceeds though the cut plane 2 to 5, the concentration profile gradually improves to uniform. As the flow reaches cut plane 5, the concentration is closely homogeneous, revealing the efficient mixing of the two different fluids.

**Figure 11:**
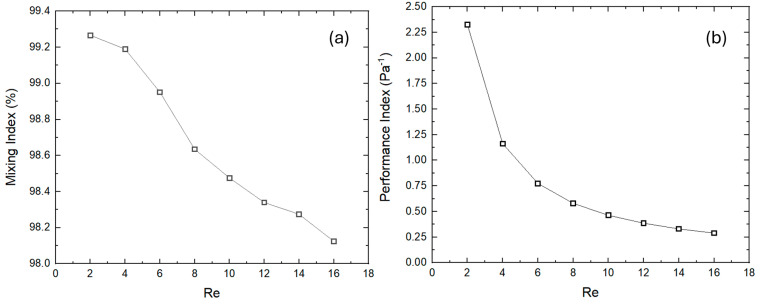
Variation in mixing index (**a**) and performance index (**b**) with different Reynolds numbers.

The assessments of the mixing index and performance index are also conducted with different Reynolds numbers. Figure 11a shows the graphical representations of mixing index and performance with different Re numbers. The value of mixing index achieved is 99.26% at a lower Re of 2, indicating higher mixing efficiency. Similarly, the mixing index gradually decreased with the increase in Re, revealing a lower mixing efficiency. The obtained value of the mixing index is 98.12 at Re 16. At higher RE values, the flow transition occurred from a diffusion regime to a convection regime, while at low Re, molecular diffusion is key in mixing phenomena, leading to the efficient mixing of fluids. In addition, at a higher Re value, the available time for diffusion also reduces, yielding a less effective mixing. On the other hand, the performance index quantifies the pressure drops within the micromixer, and the performance index decreases potentially as Re increases. The maximum and minimum values of the performance indexes equivalent to 2.32664 Pa^−1^ and 0.28918 Pa^−1^ are observed at Re 2 and 16, respectively. This implies that the pressure drop improves as the flow rate increases.

**Figure 12:**
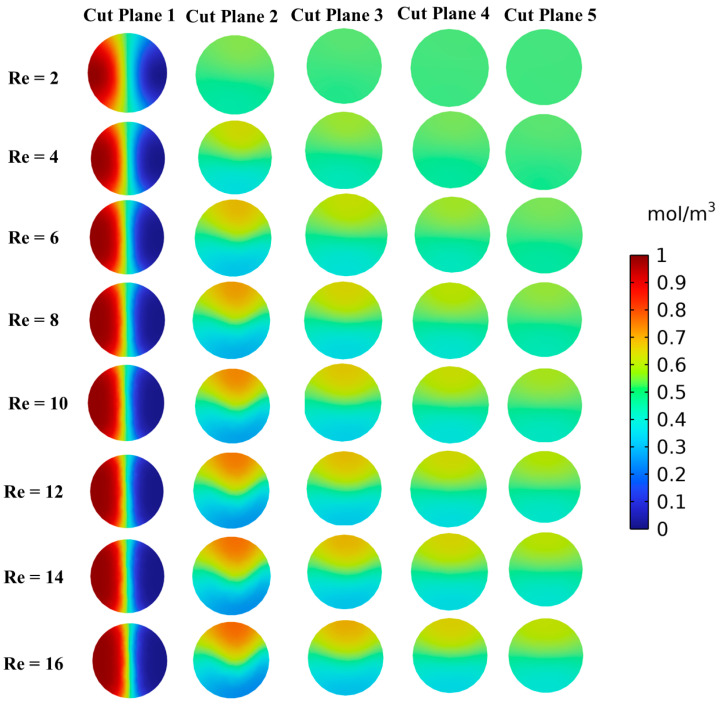
Variation in concentration at five different cut planes for different Reynolds numbers.

Furthermore, Figure 12 illustrates the concentration contours at five different cut planes along the channel length for various Reynolds numbers (Re = 2 to 16). The concentration contours exhibit a significant mixing at Re = 2, while lowest mixing is observed at Re = 16. In addition, the lower mixing is observed at cut plane 1, while higher mixing is observed at cut plane 5, indicating effective diffusion-dominated mixing within the channel. As the Re rises, the mixing efficiency gradually declines, as shown at cut plane 16. It is evident that the concentration contours exhibit a lower uniformity which can be clearly observed. The graphical representations demonstrate that mixing is more efficient at lower Re due to the molecular diffusion-dominated region, while a higher Re leads to rapid flow and reduced diffusion time, resulting in incomplete mixing.

**Figure 13:**
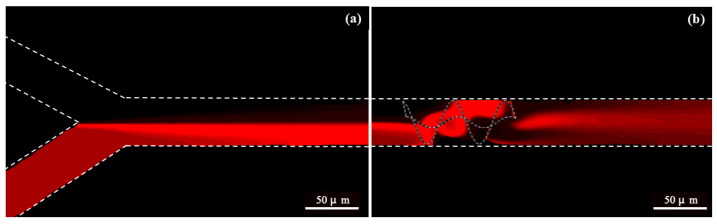
Fluorescence images of micromixer (**a**) without the integration of an Archimedes screw and (**b**) with the integration of an Archimedes screw.

Figure 13a,b represents the fluorescence images of a micromixer with and without the integration of an Archimedes screw. The channels are defined by dotted lines. It can be observed from Figure 13a that the flowing streamlines cover only half of the channel path without integration of a mixing-unit, resulting in lower mixing along the channel length. The reason behind this unmixing behavior is due to laminar flow conditions without any chaotic and diffusion mixing phenomena. Similarly, a higher mixing of fluid streamlines is observed in Figure 13b with the integration of a mixing unit. The integration of an Archimedes screw provides rotary flow, which enhance the turbulence flow, resulting in an enhancement of diffusion and chaotic mixing along the channel length. Moreover, a comparison study has also been conducted in order to compare the present work (numerical and experimental results) with those in the literature, as listed in Table 5. It can be observed that the authors evaluated the mixing performance using various kinds of passive-type microfluidics micromixers, but the proposed micromixer design integrated with an Archimedes screw has a better mixing performance equivalent to 98.47%. In addition, numerical results are in good agreement with experimental results and lie within the acceptable range.

### 3.3. Analysis of Flow Field

#### 3.3.1. Pressure Field

**Figure 14:**
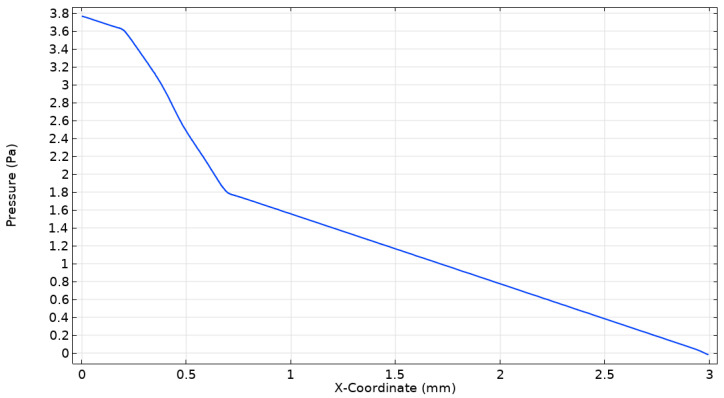
Variation in pressure along the channel length at Re = 10.

The pressure field plays a significant role in the assessment of the mixing performance of a microfluidics micromixer. Figure 14 illustrates a variation in pressure along the microchannel length at Re = 10. It can be observed that pressure drops along the microchannel length. At the junction of the channel before the mixing unit, the pressure is approximately 3.8 Pa and drops steadily as the fluid proceeds along the microchannel length, observing nearly 0 Pa at the outlet of the microchannel. Initially, the pressure drops rapidly at the junction of channels, indicating meaningful resistance due to the geometrical features of the mixing unit and resulting in good mixing. Additionally, as the flow proceeds to outlet, the pressure tends to gradually become linear, indicating smoother flow and lower resistance.

**Figure 15:**
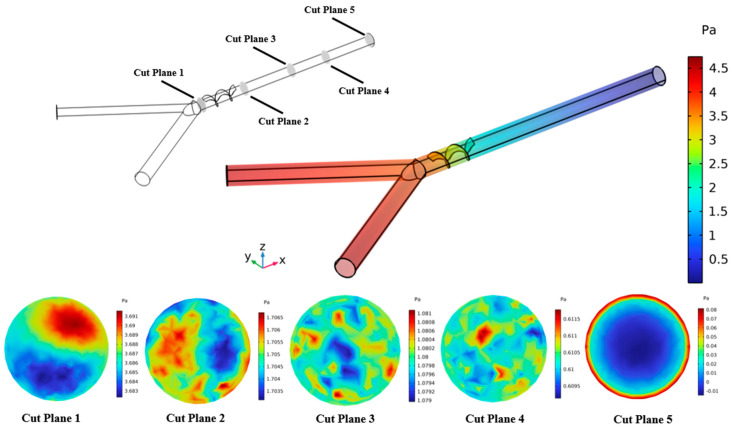
Pressure distribution within the micromixer channel at Re = 10.

Moreover, pressure distribution within the micromixer channel at different cut planes for Re = 10 is also represented in Figure 15. The pressure is observed to be higher at the inlet section, declining progressively along the channel length and observed to be significantly lower at the outlet. The variation in pressure distribution was also illustrated in a cross-sectional view for five different cut planes. The higher pressure is observed and presents a significant variation due to the resistance in flow in mixing regions. As the flow proceeds toward the outlet (cut plane 2 to 5), the pressure drops gradually, resulting in the minimal pressure distribution shown in cut plane 5.

#### 3.3.2. Velocity Field

**Figure 16:**
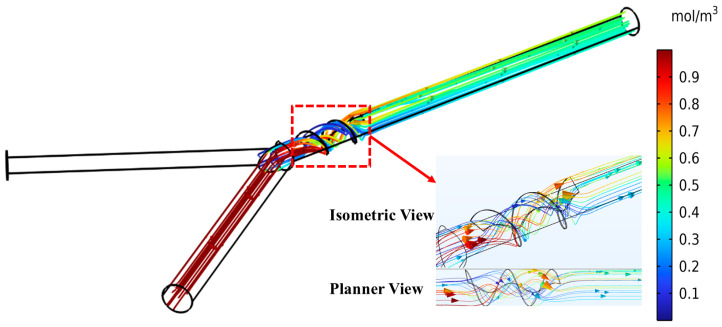
Mixing streamlines within the micromixer channel at Re = 10.

The velocity field generally describes the variation in fluid velocity while flowing through the microchannel, demonstrating the velocity magnitude and direction of flow at different cross-sections. This helps to understand how the fluid flows and interacts, showing the high and low velocity domains. In the assessment of mixing phenomena, the velocity field facilitates the identification of flow patterns, such as vortices, especially in laminar flow conditions, mainly affecting the mixing performance by improving the fluid layer interaction and advancing diffusion phenomena. Figure 16 illustrates the mixing streamlining the flow path, with noticeable twisting and turning patterns created by the integration of an Archimedes screw as a mixing unit in the micromixer channel. These patterns increase mixing by enhancing the contact between fluid layers, resulting in molecular diffusion time.

**Figure 17:**
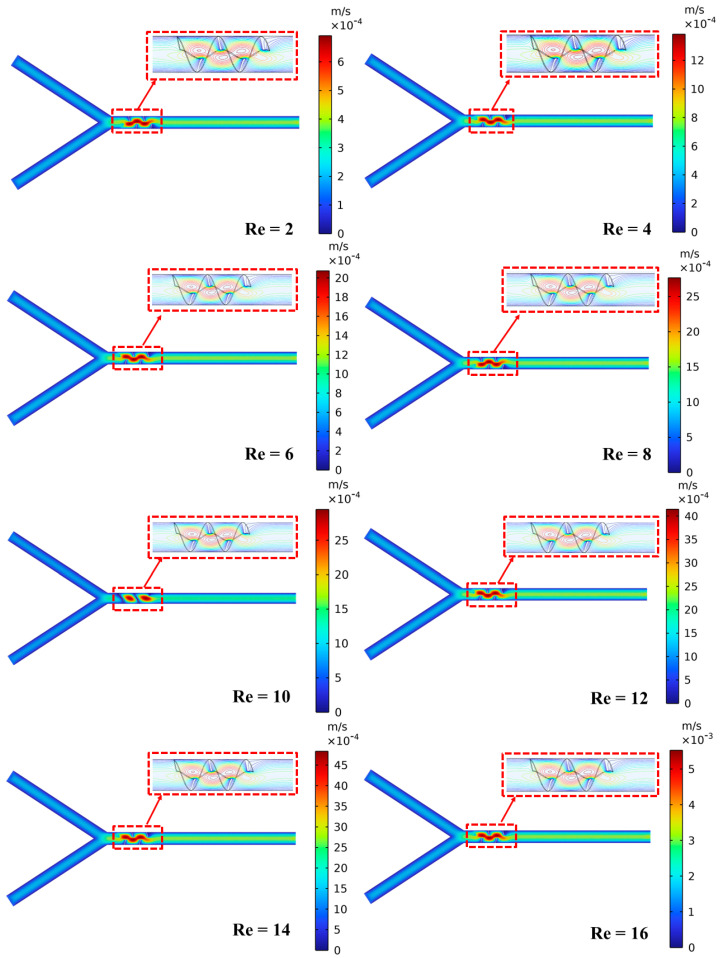
Velocity distribution in micromixer channel for different Reynolds numbers.

Moreover, the velocity field for different Reynolds numbers (Re = 2 to 16) is also illustrated in Figure 17. It can be observed that the flow is primarily in laminar conditions and the velocity field is significantly uniform at Re = 2, exhibiting minimal streamline disruption. This indicates a diffusion-dominated mixing where molecular diffusion plays a vital role in homogenizing the fluids. In addition, the velocity field becomes more assertive with an increase in Re, resulting in higher velocity magnitudes. Furthermore, the representation of flow patterns such as the cortices and twisting dynamics of the fluid flow at different Reynolds numbers are produced by the mixing unit. These complex flow patterns increase the fluid layer interaction and enhance mixing through advection and chaotic flow dynamics.

## 4. Conclusions

This paper presented a new kind of micromixer integrated with an Archimedes screw in a circular Y-shape channel aiming to achieve a higher mixing index and performance index while maintaining a lower pressure drop. First, the geometrical parameters such as screw length (*l*), screw pitch (*p*) and gap (*s*) between the screw and the inner wall of the channel are optimized using a DoE approach, and numerical simulations for each experimental run are performed using COMSOL Multiphysics. The obtained optimal design is then scaled up and fabricated using a 3D additive manufacturing approach. In addition, the optimal design is then numerically and experimentally investigated at different Reynolds numbers to explore the mixing performance. The detailed conclusions extracted from this study are described below.

1. The integration of an Archimedes screw in a circular Y-shape micromixer has a positive influence on mixing performance. In addition, the Design of Experiment (DoE) with Central Composite Design (CCD) is observed as a competent approach for the optimization of the mixing unit.
2. The optimal geometrical parameters of a screw length of 0.5 mm, screw pitch of 0.23409 mm and a gap of 0.004 deliver the best result compared to other designs. The achieved values of the mixing index and performance index are 98.47% and 20.15 Pa^−1^, respectively.
3. In addition, the experimental and numerical investigation of the optimal micromixer design is also performed at different Reynolds numbers. It is evident that the mixing index and performance index decrease gradually with the increase in Reynolds numbers. This is because of less contact time allowed for diffusion, yielding less effective mixing. The maximum and minimum value of the mixing indexes are recorded at Reynolds numbers of 2 and 16, respectively.
4. In addition, the pressure field and velocity field are also examined at various Reynolds numbers. It is observed that pressure drops nonlinearly along the length of the channel due to the resistance of the mixing unit. After mixing, the pressure drops gradually and almost reaches 0 Pa. Moreover, a higher velocity is observed at the central portion of channel and a minimum at the wall of the channel.
5. Furthermore, the comparison study is also carried out to compare the present work with the current literature. It is evaluated that the proposed micromixer design integrated with an Archimedes screw has a better mixing performance of 98.47% at Renolds numbers of 10. In addition, the numerical simulation results obtained are in good agreement with experimental results and keep in acceptable range.
6. Based on the current research work, further studies such as the investigation of rheological properties and wet chemistry analysis for various Newtonian and Non-Newtonian fluids under acoustic and non-acoustic conditions are required to examine the dynamic behavior of fluids flowing through a microchannel and integrated with an Archimedes screw. In addition, the impact of various blade inclination angles on mixing performance must also be analyzed. Moreover, the structural behavior of the Archimedes screw can also be studied to evaluate its structural stability and strength under various operating parameters.

## Figures and Tables

**Table 1:** Summary of various micromixers design.

Year	Objective	Micromixer Shape	Actuation Type	Microchannel Design	References
2024	Development and investigation of passive-type oscillating micromixer to examine mixing and fluid flow characteristics	Oscillating micromixer with impinging jets	passive	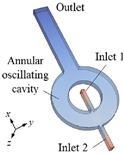	[51]
2024	Conducted mixing times analysis for T shape and V shape micromixer for Raynolds number ranging up to 1500	T-shape and V-shape micromixer	Passive	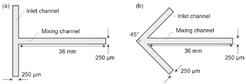	[52]
2024	Investigation of mass transfer and mixing behavior of jet-to-counter flow micromixer	Jet-to-counter flow type micromixer	Passive	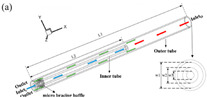	[53]
2024	Development and investigation of Y-shape acoustic inertial micromixers using perturbation theory and the Generalized Lagrangian Mean (GLM) theory	Y-shape acoustic-inertial micromixer	Active	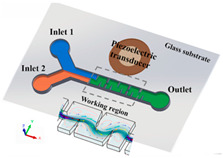	[7]
2023	Development and characterization of 3D zig-zag microchannel.	3D Zig-Zag microchannel	Passive	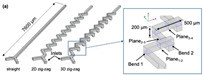	[54]
2023	Evaluation of mixing performance and fluid flow characterization in fractal tree-type microchannels.	Fractal tree-type micromixer	Passive	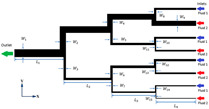	[47]
2022	Design, optimization and investigation of plat column tray inspired micromixer for organic synthesis	Plat column tray-inspired micromixer	Passive	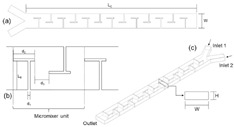	[55]
2022	Design and investigation of innovative flower-shape sharp-edge acoustic micromixer	Flower-shape acoustic micromixer	Active	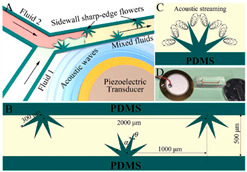	[56]
2021	Investigation and performance analysis of spiral-shape micromixer for efficient mixing	Spiral-shape micromixer	Passive	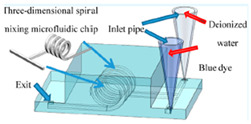	[57]
2020	Investigation of serpentine micromixer for the activation of amino acid monomers	Serpentine-shape micromixer	Passive	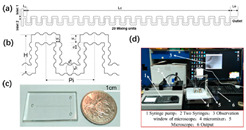	[58]

**Table 2:** Independent variables and their corresponding levels for the optimization of the micromixer.

Independent Variable	Symbols	Coded Levels				
		−α	−1	0	+1	+α
Length of screw (mm)	*l*	0.163641	0.3	0.5	0.7	0.836359
Pitch of screw (mm)	*p*	0.0659104	0.1	0.15	0.2	0.23409
Gap (mm)	*s*	0.000636414	0.002	0.004	0.006	0.00736359

**Table 3:** Description of mesh independence study.

Mesh Refinement Level	Number of Elements	Number of Nodes	Mixing Index
1	13,150	4449	0.94897
2	18,221	6078	0.95991
3	32,334	10,113	0.96278
4	71,741	20,607	0.96388
5	148,480	39,488	0.96356
6	618,291	138,399	0.96378
7	1,557,605	333,504	0.96357

**Table 4:** Description of DoE with geometrical parameters in mm and responses.

Run	Screw Length (*l*)	Screw Pitch (*p*)	Gap (*s*)	Mixing Index (*MI*)	Pressure Drop (Δ*p*)	Performance Index (*PI*)
1	0.3	0.2	0.006	95.49	3.41	27.97
2	0.5	0.0659	0.004	95.74	17.54	5.45
3	0.3	0.1	0.002	85.26	16.11	5.92
4	0.5	0.15	0.004	95.37	11.48	8.3
5	0.7	0.1	0.002	94.33	43.03	2.19
6	0.3	0.2	0.002	95.42	3.43	27.74
7	0.5	0.15	0.0073	96.46	11.27	8.46
8	0.7	0.2	0.006	96.03	9.35	10.26
9	0.3	0.1	0.006	85.34	15.71	5.43
10	0.7	0.1	0.006	94.28	41.89	2.25
11	0.5	0.2340	0.004	98.47	4.88	20.15
12	0.5	0.15	0.004	95.36	11.49	8.29
13	0.7	0.2	0.002	95.99	9.44	10.16
14	0.8363	0.15	0.004	89.9	20.37	4.41
15	0.5	0.15	0.004	95.37	11.48	8.3
16	0.5	0.15	0.0006	95.26	11.60	8.2
17	0.1636	0.15	0.004	95.23	2.46	38.7

**Table 5:** Comparison study with current literature.

Flow Rates/Re	Mixing Index	References
10–70	0.90	[25]
0.1–10	0.88	[31]
0.3 µL/min	0.90	[64]
0.1 to 100	0.85	[65]
2 mL/min	0.89	[66]
2–16	0.9847	Present study (numerical)
2–16	0.9798	Present study (experimental)

## Data Availability

The original contributions presented in the study are included in the article, further inquiries can be directed to the corresponding author.

## References

[1] Zhou Z., Zhang L., Almond H., Ge D. (2024). Investigation of mixing characteristics in a novel SAR micromixer with locally overlapping V-shaped flow channels. Chem. Eng. Process. Process Intensif..

[2] Kharaji Z.G., Bayareh M. (2024). Non-Newtonian fluid mixing in spiral micromixers: An extensive numerical analysis. Int. Commun. Heat Mass Transf..

[3] Han W., Li W., Zhang H. (2024). Insight into mixing performance of bionic fractal baffle micromixers based on Murray’s Law. Int. Commun. Heat Mass Transf..

[4] Gupta S., Sasmal C. (2024). On designing a wavy sinusoidal micromixer for efficient mixing of viscoelastic fluids harnessing elastic instability and elastic turbulence phenomena. Chem. Eng. Sci..

[5] Bazaz S.R., Sayyah A., Hazeri A.H., Salomon R., Mehrizi A.A., Warkiani M.E. (2024). Micromixer research trend of active and passive designs. Chem. Eng. Sci..

[6] Bahrami D., Nadooshan A.A., Bayareh M. (2022). Effect of non-uniform magnetic field on mixing index of a sinusoidal micromixer. Korean J. Chem. Eng..

[7] Mu S., Lu Y., Zhu G. (2024). Numerical simulation and coupling mechanism study of acoustic-inertial micromixer. Chem. Eng. J..

[8] Yu S., Jeon T.J., Kim S.M. (2012). Active micromixer using electrokinetic effects in the micro/nanochannel junction. Chem. Eng. J..

[9] Bizualem Y.D., Nurie A.G., Nadew T.T. (2024). A review on biodiesel micromixers: Types of micromixers, configurations, and flow patterns. Heliyon.

[10] Tang S.-Y., Sivan V., Petersen P., Zhang W., Morrison P.D., Kalantar-Zadeh K., Mitchell A., Khoshmanesh K. (2014). Liquid Metal Actuator for Inducing Chaotic Advection. Adv. Funct. Mater..

[11] Nouri D., Zabihi-Hesari A., Passandideh-Fard M. (2017). Rapid mixing in micromixers using magnetic field. Sens. Actuators A Phys..

[12] Huang C., Tsou C. (2014). The implementation of a thermal bubble actuated microfluidic chip with microvalve, micropump and micromixer. Sens. Actuators A Phys..

[13] Yanbo M., Sun C.-P., Fields M., Haake D., Churchill B., Ho C.-M. (2008). An unsteady microfluidic T-form mixer perturbed by hydrodynamic pressure. J. Micromech. Microeng..

[14] Huang P.-H., Zhao S., Bachman H., Nama N., Li Z., Chen C., Yang S., Wu M., Zhang S.P., Huang T.J. (2019). Acoustofluidic Synthesis of Particulate Nanomaterials. Adv. Sci..

[15] Zhao S., Huang P.-H., Zhang H., Rich J., Bachman H., Ye J., Zhang W., Chen C., Xie Z., Tian Z. (2021). Fabrication of tunable, high-molecular-weight polymeric nanoparticlesviaultrafast acoustofluidic micromixing. Lab Chip.

[16] Bai C., Zhou W., Yu S., Zheng T., Wang C. (2022). A surface acoustic wave-assisted micromixer with active temperature control. Sens. Actuators A Phys..

[17] Modarres P., Tabrizian M. (2020). Phase-controlled field-effect micromixing using AC electroosmosis. Microsyst. Nanoeng..

[18] Wang J., Feng Q., Yao J., Zhao K. (2023). Insights into a T-type micromixer with novel electromagnetic mixing. Int. J. Heat Mass Transf..

[19] Endaylalu S.A., Tien W.H. (2022). A Numerical Investigation of the Mixing Performance in a Y-Junction Microchannel Induced by Acoustic Streaming. Micromachines.

[20] Jalili H., Raad M., Fallah D.A. (2020). Numerical study on the mixing quality of an electroosmotic micromixer under periodic potential. Proc. Inst. Mech. Eng. Part C J. Mech. Eng. Sci..

[21] Gong Y., Cheng X. (2023). Numerical investigation of electroosmotic mixing in a contraction–expansion microchannel. Chem. Eng. Process. Process Intensif..

[22] Mondal B., Mehta S.K., Pati S., Patowari P.K. (2021). Numerical analysis of electroosmotic mixing in a heterogeneous charged micromixer with obstacles. Chem. Eng. Process. Process Intensif..

[23] Barman C., Bandopadhyay A. (2023). Mixing intensification in an acoustofluidic micromixer aided with micro-pillars. Chem. Eng. Process. Process Intensif..

[24] Juraeva M., Kang D.J. (2020). Mixing performance of a cross-channel split-and-recombine micro-mixer combined with mixing cell. Micromachines.

[25] Afzal A., Kim K.Y. (2012). Passive split and recombination micromixer with convergent-divergent walls. Chem. Eng. J..

[26] Mouza A.A., Patsa C.M., Schönfeld F. (2008). Mixing performance of a chaotic micro-mixer. Chem. Eng. Res. Des..

[27] Tripathi E., Patowari P.K., Pati S. (2021). Comparative assessment of mixing characteristics and pressure drop in spiral and serpentine micromixers. Chem. Eng. Process. Process Intensif..

[28] Hardt S., Pennemann H., Schönfeld F. (2006). Theoretical and experimental characterization of a low-Reynolds number split-and-recombine mixer. Microfluid. Nanofluidics.

[29] Nimafar M., Viktorov V., Martinelli M. (2012). Experimental Investigation of Split and Recombination Micromixer in Confront with Basic T- and O- type Micromixers. Int. J. Mech. Appl..

[30] Wang Z., Yan X., Zhou Q., Wang Q., Zhao D., Wu H. (2023). A Directly Moldable, Highly Compact, and Easy-for-Integration 3D Micromixer with Extraordinary Mixing Performance. Anal. Chem..

[31] Tripathi E., Patowari P.K., Pati S. (2021). Numerical investigation of mixing performance in spiral micromixers based on Dean flows and chaotic advection. Chem. Eng. Process. Process Intensif..

[32] Ward K., Fan Z.H. (2015). Mixing in microfluidic devices and enhancement methods. J. Micromech. Microeng..

[33] You J.B., Kang K., Tran T.T., Park H., Hwang W.R., Kim J.M., Im S.G. (2015). PDMS-based turbulent microfluidic mixer. Lab Chip.

[34] Xiong M., Yang J., Ding X., Li H., Zhang H. (2023). Topology optimization design of micromixer based on principle of Tesla valve: An experimental and numerical study. Chem. Eng. Process. Process Intensif..

[35] Zou L., Gong Y., Chen L., Yi X., Liu W. (2021). Design and evaluation of two-dimensional passive micromixer based on unbalanced convergence-divergence-splits and reverse-collisions-recombination. Chem. Eng. Sci..

[36] Rahmannezhad J., Mirbozorgi S.A. (2019). CFD analysis and RSM-based design optimization of novel grooved micromixers with obstructions. Int. J. Heat Mass Transf..

[37] Khoshdast H., Shojaei V., Khoshdast H. (2017). Combined application of computational fluid dynamics (CFD) and design of experiments (DOE) to hydrodynamic simulation of a coal classifier. Int. J. Min. Geo. Eng..

[38] Lira J.O.B., Riella H.G., Padoin N., Soares C. (2020). CFD + DoE optimization of a flat plate photocatalytic reactor applied to NOx abatement. Chem. Eng. Process. Process Intensif..

[39] Santana H.S., Silva J.L., da Silva A.G.P., Rodrigues A.C., de Lima Amaral R., Noriler D., Taranto O.P. (2021). Development of a new micromixer elis for fluid mixing and organic reactions in millidevices. Ind. Eng. Chem. Res..

[40] Ortega-Casanova J. (2017). Application of CFD on the optimization by response surface methodology of a micromixing unit and its use as a chemical microreactor. Chem. Eng. Process. Process Intensif..

[41] Nguyen T.Q., and Park W.T. (2020). Fabrication method of multi-depth circular microchannels for investigating arterial thrombosis-on-a-chip. Sens. Actuators B Chem..

[42] Gaso P., Jandura D., Figurova M., Pudis D. Fabrication technology of PDMS based cylindrical and structured microchannels for LOC. Proceedings of the 12th Int. Conf. ELEKTRO 2018, 2018 ELEKTRO Conf. Proc..

[43] Uehara K., Hori Y., Ishigure T. (2023). Fabrication of Circular Cross-Section Microchannels with 3-D Lattice Arrangement and Their Use as On-Off Valves. Micro.

[44] Nguyen T.Q., Lee S.Y., Park W.T. Novel circular microchannels fabrication method for artery thrombosis investigation. Proceedings of the 2018 IEEE Micro Electro Mechanical Systems (MEMS).

[45] Lee C., Chang C.L., Wang Y.N., and Fu L.M. (2011). Microfluidic mixing: A review. Int. J. Mol. Sci..

[46] Gharib G., Bütün İ., Muganlı Z., Kozalak G., Namlı İ., Sarraf S.S., Ahmadi V.E., Toyran E., van Wijnen A.J., Koşar A. (2022). Biomedical Applications of Microfluidic Devices: A Review. Biosensors.

[47] Khan M.A., Suhaib M., Ansari M.A. (2023). Investigations on fluid flow and mixing in fractal tree like biomimetic microchannel based on Murray’s law. Chem. Eng. Process. Process Intensif..

[48] Dong X., Yaji K., Liu X. (2022). Optimum design of micromixer for a non-Newtonian fluid by topology optimization. Chem. Eng. J..

[49] Waqas M., Janusas G., Naginevičius V., Palevicius A. (2024). The Design and Investigation of Hybrid a Microfluidic Micromixer. Appl. Sci..

[50] Yang A., Chuang F., Chen C., Lee M., Chen S., Su T. (2015). A high-performance micromixer using three-dimensional Tesla structures for bio-applications. Chem. Eng. J..

[51] Yang L., Xu F., Chen G. (2024). Effective mixing in a passive oscillating micromixer with impinging jets. Chem. Eng. J..

[52] Asano S., Kudo S., Hayashi J.I. (2024). Chaotic-flow-driven mixing in T- and V-shaped micromixers. Chem. Eng. J..

[53] Ruan D., Cheng Y., Hou J., Xue S., Yang S., Ma X. (2024). Mass transfer and mixing performance in jet-to-counterflow micromixer. Chem. Eng. J..

[54] Lee B., Kim M., Oh S., Lee D.B., Lee S.-G., Kim H.M., Kim K.H., Song J., Lee C.-S. (2023). Characterization of passive microfluidic mixer with a three-dimensional zig-zag channel for cryo-EM sampling. Chem. Eng. Sci..

[55] Santana H.S., Haddad V.A., Calvo P.V.C., Palma M.S.A., da Silva A.G.P., Noriler D., Taranto O.P., Silva J.L. (2022). Design, optimization and scale-up of a new micromixer design based on plate column for organic synthesis. Chem. Eng. J..

[56] Zhao X., Chen H., Xiao Y., Zhang J., Qiu Y., Wei J., Hao N. (2022). Rational design of robust flower-like sharp-edge acoustic micromixers towards efficient engineering of functional 3D ZnO nanorod array. Chem. Eng. J..

[57] Wang J., Chen X., Liu H., Li Y., Lang T., Wang R., Cui B., Zhu W. (2022). Efficient Mixing of Microfluidic Chip with a Three-Dimensional Spiral Structure. ACS Omega.

[58] Shi H., Nie K., Dong B., Chao L., Gao F., Ma M., Long M., Liu Z. (2020). Mixing enhancement via a serpentine micromixer for real-time activation of carboxyl. Chem. Eng. J..

[59] Yolmeh M., Jafari S.M. (2017). Applications of Response Surface Methodology in the Food Industry Processes. Food Bioprocess Technol..

[60] Ponte G., Fampa M., Lee J. (2023). Computing D-Optimal solutions for huge-scale linear and quadratic response-surface models. arXiv.

[61] Bruus H. (2011). Acoustofluidics 1: Governing equations in microfluidics. Lab Chip.

[62] Bruus H., Laurell T., Lenshof A. (2014). Governing Equations in Microfluidics. Microscale Acoustofluidics.

[63] Chapter 18. Theory, 0, pp. 1–52, 2002. http://www.ce.utexas.edu/prof/Novoselac/classes/ARE372/handouts/CFD_theory.pdf.

[64] Shin C.S., Baldeck P.L., Nie Y.-M., Lee Y.-H., Lin Z.-D., Chiang C.-C., Lin C.-L. (2021). Design and evaluation of a 3D multi-manifold micromixer realized by a double-Archimedes-screw for rapid mixing within a short distance. J. Taiwan Inst. Chem. Eng..

[65] Jiang Y., Zhang Y. High performance micromixers by 3D printing based on split-and-recombine modules and twisted-architecture microchannel. Proceedings of the 5th International Conference on Intelligent Human Systems Integration: Integrating People and Intelligent Systems.

[66] Wang J., Cui B., Liu H., Chen X., Li Y., Wang R., Lang T., Yang H., Li I., Pan H. (2022). Tesla Valve-Based Flexible Microhybrid Chip with Unidirectional Flow Properties. ACS Omega.

